# Potential Roles for Probiotics in the Treatment of COVID-19 Patients and Prevention of Complications Associated with Increased Antibiotic Use

**DOI:** 10.3390/antibiotics10040408

**Published:** 2021-04-09

**Authors:** Ravina Kullar, Stuart Johnson, Lynne V. McFarland, Ellie J. C. Goldstein

**Affiliations:** 1 Expert Stewardship, Inc., 320 Superior Avenue, Newport Beach, CA 92663, USA; 2 Hines VA Hospital and Loyola University Medical Center, Chicago, IL 60141, USA; stuart.johnson2@va.gov; 3 Department of Medicinal Chemistry, University of Washington, Seattle, WA 98195, USA; mcfarland.lynne.v@gmail.com; 4 RM Alden Research Laboratory and the David Geffen School of Medicine at UCLA, Los Angeles, CA 90230, USA; ejcgmd@aol.com

**Keywords:** COVID-19, SARSCoV2, *Clostridioides difficile*, antibiotics, AAD, probiotics, antibiotic stewardship

## Abstract

Medical care for patients hospitalized with COVID-19 is an evolving process. Most COVID-19 inpatients (58–95%) received empiric antibiotics to prevent the increased mortality due to ventilator-associated pneumonia and other secondary infections observed in COVID-19 patients. The expected consequences of increased antibiotic use include antibiotic-associated diarrhea (AAD) and *Clostridioides difficile* infections (CDI). We reviewed the literature (January 2020–March 2021) to explore strategies to reduce these consequences. Antimicrobial stewardship programs were effective in controlling antibiotic use during past influenza epidemics and have also been shown to reduce healthcare-associated rates of CDI. Another potential strategy is the use of specific strains of probiotics shown to be effective for the prevention of AAD and CDI prior to the pandemic. During 2020, there was a paucity of published trials using these two strategies in COVID-19 patients, but trials are currently ongoing. A multi-strain probiotic mixture was found to be effective in reducing COVID-19-associated diarrhea in one trial. These strategies are promising but need further evidence from trials in COVID-19 patients.

## 1. Introduction

In the year since the first case of coronavirus disease (COVID-19) was reported in Wuhan, China, the pandemic has exploded worldwide, with over 111 million COVID-19 cases and over 2.5 million deaths reported as of February 2021 [1]. The pandemic has impacted the world on an unprecedented scale, burdening social, economic and healthcare systems. Lockdown measures instituted in countries during the early stages of the COVID-19 pandemic prevented an estimated 3.1 million deaths in Europe and 61 million deaths in six selected countries around the world, but the pandemic has continued despite these measures [2,3]. Relaxing control measures in many countries has led to increases in new COVID-19 cases [4]. Globally, 20% of COVID-19 patients have been hospitalized with severe acute respiratory distress, fever or sepsis, and 2–50% also have diarrhea at admission [5,6]. High rates of mortality (16–46%) due to sepsis, respiratory failure, or ventilator-associated pneumonia (VAP) have been observed in COVID-19 inpatients [7,8,9]. Most (58–95%) COVID-19 inpatients have been placed on empiric antibiotics to prevent ventilator-associated pneumonia (VAP) and secondary infections, creating an inherent challenge for antimicrobial stewardship programs (ASP) [7,10]. The justification for the high use of empiric antibiotics has been questioned based on the low rate of co-infections at admission (3–6%), the low rate of secondary bacterial and fungal infections developing during hospitalization (4–14%), and concerns about the complications of the overuse of antibiotics [7,10,11,12,13,14,15,16]. Patients admitted with COVID-19 continue to receive empiric antibiotics (58–95%), despite concerns of antibiotic overuse [10].

This increased use of antibiotics has led to concerns relating to complications associated with antibiotic use, including antibiotic-associated diarrhea (AAD) and *Clostridioides difficile* infections (CDI), the development of allergies or chronic inflammatory bowel disease and the potential for the development of antibiotic resistant bacterial strains [15,17]. Altered intestinal microbiomes have been detected in COVID-19 patients, which may make them more susceptible to AAD or opportunistic pathogens such as *C. difficile* [18,19].

Two potential strategies suggested to reduce antibiotic complications in COVID-19 patients have included antibiotic stewardship programs to reduce the overuse of empiric antibiotics and the use of specific probiotics to prevent AAD or CDI [10,20]. Probiotics have been previously shown to effectively reduce AAD and CDI among other types of diseases, due to multiple mechanisms of action found in some probiotic strains, including destruction of pathogenic toxins, inference with pathogen attachment to host cells, and the ability to act as an immune regulator among other mechanisms [21,22,23,24].

Our paper raises awareness of these issues, and reviews the potential strategies for the following: (1) antimicrobial stewardship programs (ASP) for COVID-19 patients; (2) potential for *C. difficile* infections or AAD in subsequent waves of COVID-19; (3) use of probiotics to avert collateral damage associated with increased antibiotic use; (4) challenges involved in probiotic use including shifts in taxonomy and Lactobacillus susceptibilities to antibiotics; (5) use of probiotics to treat COVID-19-associated diarrhea.

## 2. Antimicrobial Stewardship Programs (ASP) and COVID-19 Patients

The initial rationale for antibiotic use in COVID-19 patients was based on experiences with bacterial superinfections in influenza patients, which was often the factor precipitating admission to intensive care units (ICU). Various studies reported initial co-infection or secondary bacterial pneumonia in 11–35% of hospitalized patients with influenza, with most of the superinfections caused by Streptococcus pneumoniae or Staphylococcus aureus [25]. However, the literature demonstrates that the same does not hold true for COVID-19, as most COVID-19 patients are admitted to an ICU due to viral respiratory distress and not bacterial pneumonia [16]. The rates of co-infections at admission are also lower in COVID-19 patients. A recent systematic review and meta-analysis of 31 studies showed that only 7% of hospitalized patients with COVID-19 had evidence of bacterial co-infections, yet >90% received empirical antibiotics [26]. In another study of 989 hospitalized COVID-19 patients in Spain, only 3% had a co-infection at admission and only 4.4% developed a secondary infection while admitted (most due to Staphylococci or Pseudomonas aeruginosa) [12]. In addition, antibiotic use in COVID-19 patients has included multiple types of antibiotics and anti-fungal medications. In one study of 99 COVID-19 patients in China, 71% received antibiotics for 3–17 days, 45% received combination antibiotics and 15% were also given anti-fungal medications, but only one patient had a culture-confirmed bacterial co-infection [27].

ASPs have been effective in reducing the overuse of antibiotics in studies done before the COVID-19 pandemic and most have reduced the incidence of healthcare-associated infections [28]. A component in these programs has been to evaluate the rationale behind the use of antibiotics. Buetti et al. retrospectively reviewed 48 intubated ICU patients with COVID-19 over one month to determine if early antibiotic administration decreased mortality [29]. There was no difference in mortality in those who received antibiotics compared to those who did not (26% died with antibiotics vs. 24% died without antibiotics, *p* = 0.86). Staub et al. reported increased antibiotic use at their medical center in Tennessee USA, after COVID-19 patients began to be admitted and, in response, implemented an ASP for their COVID-19 patients [30]. A significant reduction in antibiotic use was observed after the ASP was implemented. During the spring of 2020, a hospital in New York City experienced an upsurge in admitted COVID-19 patients, which resulted in an increase in the number of patients on mechanical ventilation and a significant increase in the use of empiric antibiotics [31]. Although their staff adapted their ASP for COVID-19 patients and a reduction in antibiotic use was noted, there were many challenges ranging from increased workflow, staff shortages, limited time to review cases and lack of supplies [31].

ASP for COVID-19 patients should incorporate recent guidelines on which patients should receive empiric antibiotics. The World Health Organization (WHO) released an updated interim guidance for the clinical management of COVID-19 patients, stating that antibiotic use is dependent on the classification of the severity of COVID-19 disease [32]. The WHO recommended against antibiotic use in all patients with mild COVID-19, as well as for those with moderate COVID-19, unless there is a clinical suspicion of a bacterial infection. For moderate cases, the WHO recommended considering empiric antibiotic treatment for preventing pneumonia in the elderly, especially those in long-term care facilities, and for children <5 years old. The guidance is that antibiotics should, for the most part, be reserved for those suffering from severe COVID-19 symptoms.

ASPs can serve a role as the primary gatekeeper for the appropriate use of COVID-19 treatments to optimize antibiotic selection and to minimize misuse. ASPs can also be used to develop treatment protocols for COVID-19 patients, and then communicating them to the frontline clinicians during the pandemic. Leveraging the knowledge of the ASP team is critical in the setting of the current COVID-19 pandemic.

## 3. Prevention Antibiotic-Associated Complications in COVID-19 Patients

### 3.1. Antibiotic-Associated Diarrhea (AAD)

AAD is defined as diarrhea associated with antibiotic exposure, either while on antibiotics or for up to eight weeks after antibiotics have been discontinued [33]. Although the etiologies for AAD are varied and not all the pathogens are currently identifiable, nearly one-third of AAD cases are due to *C. difficile*. AAD occurs in approximately 20% of patients given antibiotics, but the rate varies depending upon the type of antibiotic, age, hospitalization, co-morbidities and other risk factors [33]. The consequences of AAD have included extended hospital stays, higher mortality rates and higher costs of healthcare [33]. Unfortunately, in the published reports describing the clinical courses of COVID-19 patients, few studies were found that documented the rates of AAD. However, as it is a well-documented outcome of antibiotic exposure, this outcome should be considered in the treatment paradigm of COVID-19 patients.

### 3.2. C. difficile Infections (CDI)

*C. difficile* is the leading cause of healthcare associated gastrointestinal infections and was responsible for ~460,000 cases during 2017 with a 5.2% attributable mortality and costs of approximately USD 5 billion in excess medical costs [34,35]. Data are still emerging regarding the interaction of CDI and COVID-19 patients. Sandhu et al. reported nine cases of CDI in COVID-19 patients from Detroit Medical Center Hospitals [36]. Three of these patients had prior CDI episodes, two had diarrhea and were positive for *C. difficile* on admission, and seven developed CDI after a median of six days after their COVID-19 diagnosis. All these patients had received antibiotics for a mean of five days prior to the onset of CDI. The median age was 75 years and 78% were female, four patients died, and one was discharged to hospice care. While the mortality and outcomes of this small cohort were likely influenced to a large degree by their SARS-CoV-2 infection, the patients had many of the typical risk factors for CDI, including older age, hospitalization, and antibiotic exposure. The potential additional morbidity associated with CDI was highlighted by a recent case report from Spain where a 64-year-old woman presented with severe colitis caused by *C. difficile* that required a colectomy 10 days after a one-month hospital admission for COVID-19 infection with bilateral pneumonia [37]. Granata et al. also reported a retrospective case–control study of COVID-19 patients with and without CDI in eight Italian hospitals [38]. Patients with CDI had more COVID-19-associated complications and longer inpatient stays (mean of 16 days longer) compared to COVID-19 patients with no CDI. Risk factors associated with CDI among these patients included the presence of cardiovascular disease, previous hospitalization, steroids, bacterial superinfection, and antibiotic administration. The authors concluded that CDI complicates the clinical course of COVID-19 in patients, especially those with co-morbidities, previous healthcare and antibiotic exposure and urged compliance with antibiotic stewardship programs during the pandemic. Two other studies found similar rates of CDI in COVID-19 patients compared to non-COVID-19 patients. Bentivegna et al. tested 483 inpatients and reported that CDI were present in 5% of COVID-19 patients and in 3% of non-COVID-19 patients at their hospital in Italy [39]. Laszkowska et al. tested 4973 patients at two hospitals in New York and found slightly fewer CDI cases (5%) in COVID-19 patients compared to COVID-19 free patients (8%), but also noted that the rate of testing for *C. difficile* declined during the pandemic due to other priorities [40].

Given the increase in antibiotic usage during this pandemic, we had earlier postulated that we might expect a resurgence or “subsequent waves” of CDI in COVID-19 patients. Prior data suggested that there is a seasonality for CDI and that seasonal peaks follow peaks of respiratory tract virus infections [41,42,43]. This association has been noted for both influenza and respiratory syncytial virus. Gilca et al. postulated that the association of higher rates of CDI was due to increased antibiotic use for respiratory infections [42].

However, we found that recent studies reported a surprising reduction in CDI cases, even given the increased use of antibiotics for COVID-19 patients. Ochoa-Hein et al. reported a significant reduction in the number of CDI cases (1.4/10,000 patient-days) after their hospital in Mexico City was converted to an exclusive COVID-19 facility compared to year prior (9.3/10,000 patient-days) [44]. Bentivegna et al. started a COVID-19 prevention program at a hospital in Italy involving increased use of personal protective equipment, increased hand washing, enhanced surface disinfection and limitation of visitors [39]. Excluding ICU patients, a significantly lower rate of CDI (3.7/100 discharges) was observed compared to the previous year (6.6/100 discharges). Hazel et al. also reported a lower rate of CDI at their hospital in Ireland during the pandemic (2.15/10,000 bed-days) compared to the prior year (4.24/10,000 bed-days) and attributed the reduction to lower bed occupancy and enhanced infection control practices for COVID-19 patients [45]. Wee et al. reported no significant increases in CDI after they started a multimodal infection control program for their COVID-19 patients admitted to a hospital in Singapore (3.47 CDI/10,000 patient-days during the pandemic compared to 3.65/10,000 patient-days pre-pandemic) [46]. These studies may explain why the expected surge in CDI cases have not appeared during the pandemic. Enhanced infection control programs aimed at controlling the transmission of SARS-CoV-2 virus may also be effective in reducing other healthcare-associated pathogens such as *C. difficile*. However, other factors may also contribute to the lower reported rates of CDI, including lower hospital bed occupancies and a lower rate of testing for *C. difficile*. As we are in the midst of the pandemic, vigilance is warranted for future outbreaks, even as enhanced infection control programs are continued.

## 4. Potential Use of Probiotics for Antibiotic-Associated Complications

Probiotics have been studied for over 50 years for a variety of diseases, ranging from the prevention of allergies and the prevention of a variety of intestinal diseases, to the treatment of acute and chronic gastrointestinal diseases and the treatment of cancer. Probiotics are the most effective in situations where the normally protective microflora has been disrupted [22,23,24,47]. Probiotics are defined as living microbes (bacteria or yeast) that may possess one or more diverse mechanisms-of-action, including interference with pathogen attachment, strengthening of the barrier effect, trophic effects, destruction of toxins or immune regulation [48,49]. While many probiotics have evidence-based efficacy for the prevention of AAD and CDI, few studies have been published in COVID-19 patients. The use of probiotics in COVID-19 patients has been suggested by many investigators, based on the ability of specific probiotics to regulate the immune response (perhaps to calm the “cytokine storm”), or to prevent other types of respiratory infections, including influenza and ventilator-associated pneumonia and to prevent AAD and CDI [50,51,52]. We thus propose that some probiotics with efficacy for AAD and CDI might be potential therapies in COVID-19 patients, preventing the consequences of the heavy use of antibiotics in these patients.

### 4.1. Probiotic Candidates for Antibiotic-Associated Diarrhea (AAD)

As up to 95% of hospitalized COVID-19 patients are receiving empiric antibiotics to prevent secondary bacterial infections, thus an increase in diarrheal rates might be expected in these patients [53]. Sniffen et al. reviewed 249 randomized controlled trials of the 29 most common probiotic types with at least two RCTs per type and provided recommendations for choosing an appropriate probiotic, accounting for both disease-specificity and strain-specificity [22]. Strong evidence for probiotic efficacy was defined when there was a net of at least two more RCTs with significant efficacy compared to trials with non-significant findings. As shown in Table 1, three probiotics were found to have strong evidence for the prevention of AAD: *Saccharomyces boulardii* CNCM I-745 “Florastor”, a mixture of three *Lactobacilli* strains (*L. acidophilus* CL1285, *L. casei* LBC80R, *L. rhamnosus* CLR2, “Bio-K+”) and *L. casei* DN114001 “Actimel”. Using meta-analysis to pool efficacy outcomes across trials may result in increased power and the ability to detect additional probiotics that may be potentially effective in COVID-19 patients. Updating data from several meta-analyses and databases found five single-strain probiotics (see Supplementary Figure S1) and four different multi-strained probiotic mixtures (see Supplementary Figure S2) that significantly prevented AAD [21,22,54,55,56].

### 4.2. Probiotic Candidates for Clostridioides difficile Infections (CDI)

In addition to ASPs, the additional use of probiotics has been suggested for the prevention of CDI in hospitalized patients [59]. A review of RCTs for the primary prevention of CDI has shown that several types of probiotic strains are effective (Table 1), but the evidence was ranked as weak to moderate. These trials designed to determine probiotic efficacy for the prevention of AAD, suffer from a lack of power, as CDI was an infrequent secondary outcome A method to overcome the lack of statistical power is to use meta-analysis. A meta-analysis of pooled data from 23 trials found that four probiotics were effective for the primary prevention of CDI: *S. boulardii* CNCM I-745, *L. casei* DN114001, a three-strain mixture of *L. acidophilus* CL1285, *L. casei* LBC80R and *L. rhamnosus* CLR2 and a two-strain mixture of *L. acidophilus* and *Bifidobacterium bifidum* [60]. The 2020 American Gastroenterology Association guidelines for the prevention of CDI has recognized the importance of probiotic strain-specificity and now recommends only four types of probiotics for primary prevention of CDI: *S. boulardii* CNCM I-745 (based on nine RCTs), the mixture of *L. acidophilus* CL1285, *L. casei* LBC80R and *L. rhamnosus* CLR2 “Bio-K+” (based on three RCTs) and two mixtures of *L. acidophilus*, *Bifidobacterium bifidum*, *Lactobacillus delbrueckii* subsp. *bulgaricus* (with or without *Streptococcus salivarius* subsp. *thermophilus*), but these last two probiotic mixtures are based on only one RCT each and lack a second confirmatory trial [61]. It should be noted that the third strain found in “Bio-K+” (*Lactoicaseibacillius rhamnosus* CLR2) was identified after the three trials were published but the strain was always present in the probiotic mixture [49].

Efficacy found in rigorously controlled trials might not translate into real-life scenarios, as factors not considered in randomized trial protocols may influence the efficacy. The successful implementation of a new procedure, staff confidence or belief in efficacy, compliance, or other healthcare practices may interfere with the effectiveness of a probiotic in a healthcare setting. Several quasi-experimental studies have been done, which compared hospital-wide CDI rates before and after a new probiotic was administered. Typically, a new probiotic strain or mixture is offered to all at-risk patients (that is, inpatients receiving new antibiotics) at a healthcare facility and CDI rates documented. One such study was done at an acute care hospital in California and an associated long-term care facility when an increase in CDI was detected [62]. Over a period of three years, three interventions were started: (1) two probiotics (*S. boulardii* CNCM I-745, “Florastor”and a two-strain mixture of *L. gasseri* and *L. helveticus*, “Lactinex”) were offered to inpatients beginning a new course of antibiotics, (2) then an ASP was started seven months later and (3) then three months later, efforts to reduce proton-pump inhibitor use was started. The result of these three measures was a 75% reduction in CDI rates in both institutions. Then, the probiotics were replaced with another type of probiotic (a three-strain mixture of *L. acidophilus* CL1285, *L. casei* LBC80R and *L. rhamnosus* CLR2, “Bio-K+”) found to be effective in controlling CDI rates in hospitals in Canada, which further reduced CDI at these facilities and this CDI reduction was sustained as long as the probiotic mixture was given [62]. The combination of ASP, infection control practices and use of various probiotics has been reported to be an effective strategy in controlling CDI [59,63].

Once an effective probiotic strain or mixture is selected, several factors that are important in the administration of the probiotic need to be considered [21,22]. The efficacy of probiotics has been found to be higher when the probiotic administration is started within 24 h of antibiotic initiation. The probiotic strain(s) should be given during the antibiotic treatment and then the probiotic should be continued for at least two weeks post-antibiotic to allow the probiotic to assist in the recovery of the normally protective microbiome. Most meta-analyses have found that a daily dose of probiotics of ≥10^9^–10^10^ microbes/day is most effective [54,55,56].

### 4.3. Probiotic Candidates for Prevention of Ventilator-Associated Pneumonia (VAP)

Nearly 33% of COVID-19 inpatients with severe acute respiratory distress are admitted to an ICU and 31–79% receive mechanical ventilation [7,30,64]. COVID-19 patients on mechanical ventilation frequently develop VAP (~31%) [7,8]. Although no RCTs testing probiotics have been performed on COVID-19 patients on ventilators, several types of probiotics have been tested in non-COVID-19 patients. Unfortunately, the data are sparse, as most types of probiotics have only one trial and lack confirmatory trials. Su et al. conducted a meta-analysis and found no significant efficacy for *L. plantarum* 299v (from two trials) but found a significant reduction in VAP when a synbiotic mixture was used (*Leuconostoc mesenteroides* 32-77:1, *L. paracasei* 19, *L. plantarum* 2362, *Pediococcus pentoseceus* 5-33:3 and inulin) with a pooled relative risk from the four trials of RR = 0.69, 95% confidence interval 0.52, 0.92) [57].

### 4.4. Challenges for Choosing the Appropriate Probiotic

In a literature review, we found over 893 RCTs spanning 59 different types of disease indications [22]. Recently, research has determined that not all probiotics are equally effective, and the efficacy is both disease-specific and strain-specific [21]. The choice of the optimal probiotic is challenging, as there are over 260 different types of probiotics available as of 2020 and the literature can be confusing. The degree of regulatory oversight differs by country, as some European countries consider probiotics as prescription medications and are tightly regulated, whereas, in the United States, most probiotics are available as dietary supplements, which are not as tightly regulated by the Food and Drug Administration (FDA). As a result, the quality of probiotic products may vary. Kolacek et al. found that up to 33% of the commercial probiotics did not contain what was listed on the label [65]. Another study tested six probiotic products produced by manufacturers with established Good Manufacturing Practices and found the labels reflected the probiotic contents with high accuracy [66]. The biggest challenge to the general public in the U.S. is that probiotics are dietary supplements, which cannot state on the label that they cure or treat a disease, despite the availability of numerous randomized, controlled trials that have been done to support their efficacy.

Another challenge when choosing an appropriate probiotic is the evolving taxonomy of bacterial strains. The genus *Lactobacillus* was proposed in 1901 and included an extremely diverse and heterogeneous collection of facultatively anaerobic, Gram-positive, non-spore-forming rods that utilized carbohydrates fermentatively to produce lactic acid as a major end product [67]. These organisms were difficult to phenotypically differentiate and relied on molecular methods to adequately name these genetically diverse species. Recent molecular advances, especially whole genome sequencing, led to a proposal that reclassified the genus *Lactobacillus* into 26 genera and 261 species [67]. Table 2 lists some examples of the shifting nomenclature for the various *Lactobacilli* species used in common probiotic products.

When probiotics are used with antibiotics, potential drug–drug interactions must also be considered [68]. The antimicrobial susceptibility of *Lactobacilli* strains is both complex and controversial, in part related to their diverse taxonomy and difficulties in routine identification [67]. This becomes especially important when *Lactobacilli* strains are used during concomitant administration with oral antibiotics. *Lactobacillus* species are generally resistant to aminoglycosides, fluoroquinolones and metronidazole. Most *Lactobacilli* species are susceptible to macrolides, with *L. rhamnosus* as an exception. *L*. *acidophilus* is generally susceptible to penicillin and vancomycin. *L. casei, L. paracasei* and *L. rhamnosus* are generally resistant to vancomycin, cephalosporins, and carbapenems.

## 5. Probiotics and the Treatment of COVID-19 Patients

Recent evidence has demonstrated that the effect of the SARS-CoV-2 virus is not limited solely to the respiratory tract. The SARS-CoV-2 virus has been detected in the intestines of COVID-19 patients and diarrhea is one of the presenting symptoms in 2–50% of newly admitted COVID-19 patients [69,70,71,72]. The use of probiotics for the treatment of COVID-19 patients is an active area of clinical research. Of 13 trials registered with ClinicalTrials.gov, only one has been completed to date, but the results have not been published [73]. Only one study has been published to date using probiotics for the treatment of COVID-19 patients. D’Ettorre et al. enrolled 70 patients with stage III COVID disease admitted to a hospital in Rome, Italy (94% had fever and 47% had diarrhea at admission) [58]. All patients were treated with standard therapies (hydroxychloroquine, azithromycin and/or tocilizumab) and then randomly chosen patients were also given an eight-strain probiotic mixture (*Lactobacillus brevis* DSM27961, *L. acidophilus* DSM32241, *L. helveticus* DSM32242, *L. paracasei* DSM32243, *L. plantarum* DSM32244, *Streptococcus thermophilus* DSM32245, *Bifidobacterium lactis* DSM32246 and *Bifidobacterium lactis* DSM32247) for two weeks at 2.4 × 10^12^ bacteria per day. Significantly more patients with diarrhea on admission who took the probiotic mixture had their diarrhea resolved by day 3 (93%) compared to patients who did not take the probiotic mixture (5%, *p* < 0.001). Interestingly, the risk of respiratory failure was also significantly higher for those not taking the probiotic mixture (Odds Ratio = 8.6, 95% C.I. 1.6–45.0). Whether probiotics will prove helpful in treating COVID-19 disease and complications depends upon the results of ongoing clinical trials.

## 6. Materials and Methods

We searched databases (PubMed and Google Scholar) and COVID-related websites (World Health Organization and CDC) from January 2020–March 2021 to identify articles on descriptions of clinical symptoms of COVID-19 disease and treatments for COVID-19 patients. We also searched the literature for clinical trials using probiotics for complications of antibiotic use (antibiotic associated diarrhea, *C. difficile* infections) and for randomized controlled trials of probiotics for the treatment of COVID-19 infections. There were no language restrictions and articles in languages other than English were translated and reviewed.

## 7. Conclusions

As more patients hospitalized with COVID-19 receive inappropriate antibiotics, complications can be expected. More antibiotic use may translate into higher rates of AAD or CDI, but the lack of documentation in COVID-19 patients on these outcomes currently limits our conclusions. These complications may result in higher healthcare costs, longer lengths-of-hospitalization stays and places an additional burden on already stressed healthcare facilities. The use of specific probiotic strains or mixtures found effective in non-COVID-19 patients may prevent AAD, CDI and VAP in COVID-19 patients, but clinical trials are urgently needed and exploration into additional probiotic strains may be warranted. However, it should be noted that the use of probiotics should be used in conjunction with effective ASPs and enhanced infection control practices.

## Figures and Tables

**Table 1:** Potential probiotic candidates for the prevention or treatment of antibiotic-associated complications seen in COVID-19 patients.

Probiotic	Number of Randomized Controlled Trials ^1^	Strength of Evidence ^2^	References
**Prevention of AAD**			
*S. boulardii* I-745 “Florastor”	18+/9−	Strong	Szajewska [56] Sniffen [22]
*L. acidophilus* CL1285 + *L. casei* LBC80R + *L. rhamnosus* CLR2 “Bio-K+”	3+/1−	Strong	Sniffen [22]
*L. casei* DN114001 “Actimel”	2+/0−	Strong	Sniffen [22]
*L. acidophilus* La5 + *B. lactis* Bb12	3+/3−	Moderate	Sniffen [22]
*L. rhamnosus* GG “Culturelle”	3+/8−	Weak	Szajewska [55]
**Prevention of CDI**			
*S. boulardii* I-745 “Florastor”	1+/11−	Weak	Sniffen [22]
*L. rhamnosus* GG “Culturelle”	1+/4−	Weak	Sniffen [22]
*L. acidophilus* CL1285 + *L. casei* LBC80R + *L. rhamnosus* CLR2 “Bio-K+”	2+/2−	Moderate	Sniffen [22]
**Prevention of VAP**			
“Synbiotic 2000” ^3^	2+/2−	Moderate	Su [57]
**Treatment of COVID-19 Diarrhea**			
“Sivomaxx” ^4^	1+/0−	Weak	D’ettorre [58]

^1^ Number of trials with significant effect (+)/number with non-significant effect (−), ^2^ Strength of evidence: strong, at least two more significant trials compared to number of non-significant trials; moderate, similar number of significant and non-significant trials; weak, more non-significant trials, ^3^ Synbiotic 2000: Leuconostoc mesenteroides 32-77:1, L. paracasei 19, L. plantarum 2362, Pediococcus pentoseceus 5-33:3 and inulin, ^4^ Sivomaxx: Lactobacillus brevis DSM27961, L. acidophilus DSM32241, L. helveticus DSM32242, L. paracasei DSM32243, L. plantarum DSM32244, Strept. thermophilus DSM32245, Bifidobacterium lactis DSM32246 and Bifidobacterium lactis DSM32247.

**Table 2:** New and retained nomenclature of *Lactobacillus* species often used in some common probiotic preparations.

Species without Name Changes	Some Genus Names Changed to
*Lactobacillus acidophilus*	*Lacticaseibacillus casei*
*Lactobacillus delbrueckii* ssp. *bulgaricus*	*Lacticaseibacillus paracasei*
*Lactobacillus crispatus*	*Lacticaseibacillus rhamnosus*
*Lactobacillus gasseri*	*Lactiplantibacillus plantarum*
*Lactobacillus johnsonii*	*Levilactabacillus brevis*
*Lactobacillus helveticus*	*Ligilactobacillus salivarius*
	*Limosilactobacillus fermentum*
	*Limosilactobacillus reuteri*

## Supplementary Materials

The following are available online at https://www.mdpi.com/article/10.3390/antibiotics10040408/s1, Figure S1: Forest plot of single-strain probiotics for the prevention of AAD, Figure S2: Forest plot of multi-strain probiotic mixtures for the prevention of AAD.

## Abbreviations

AAD	antibiotic-associated diarrhea
ASP	antibiotic stewardship programs
Bifido.	*Bifidobacterium*
C.	*Clostridioides*
CDI	*C. difficile* infections
C.I.	confidence interval
COVID-19	coronavirus disease 2019
ICU	intensive care unit
*L. acidophilus*	*Lactobacillus acidophilus*
*L. bulgaricus*	*Lactobacillus delbrueckii ss. bulgaricus*
*L. helveticus*	*Lactobacillus helveticus*
*L. casei*	*Lacticaseibacillus casei*
*L. reuteri*	*Limosilactobacillus reuteri*
*L. rhamnosus*	*Lacticasebacillus rhamnosus*
U.S.	United States of America
RCTs	randomized controlled trials
SARS-CoV-2	severe acute respiratory syndrome coronavirus 2
S.	*Saccharomyces*
*Strept*	*Streptococcus*
VAP	ventilator-associated pneumonia
vs.	versus
WHO	World Health Organization
