# Supplementary material for: Potential Roles for Probiotics in the Treatment of COVID-19 Patients and Prevention of Complications Associated with Increased Antibiotic Use
Source: Antibiotics (Basel). 2021 Apr 9;10(4):408. doi: 10.3390/antibiotics10040408 (PMC8070357; doi:10.3390/antibiotics10040408)
Supplement: Supplementary file 1 [file antibiotics-10-00408-s001.pdf]

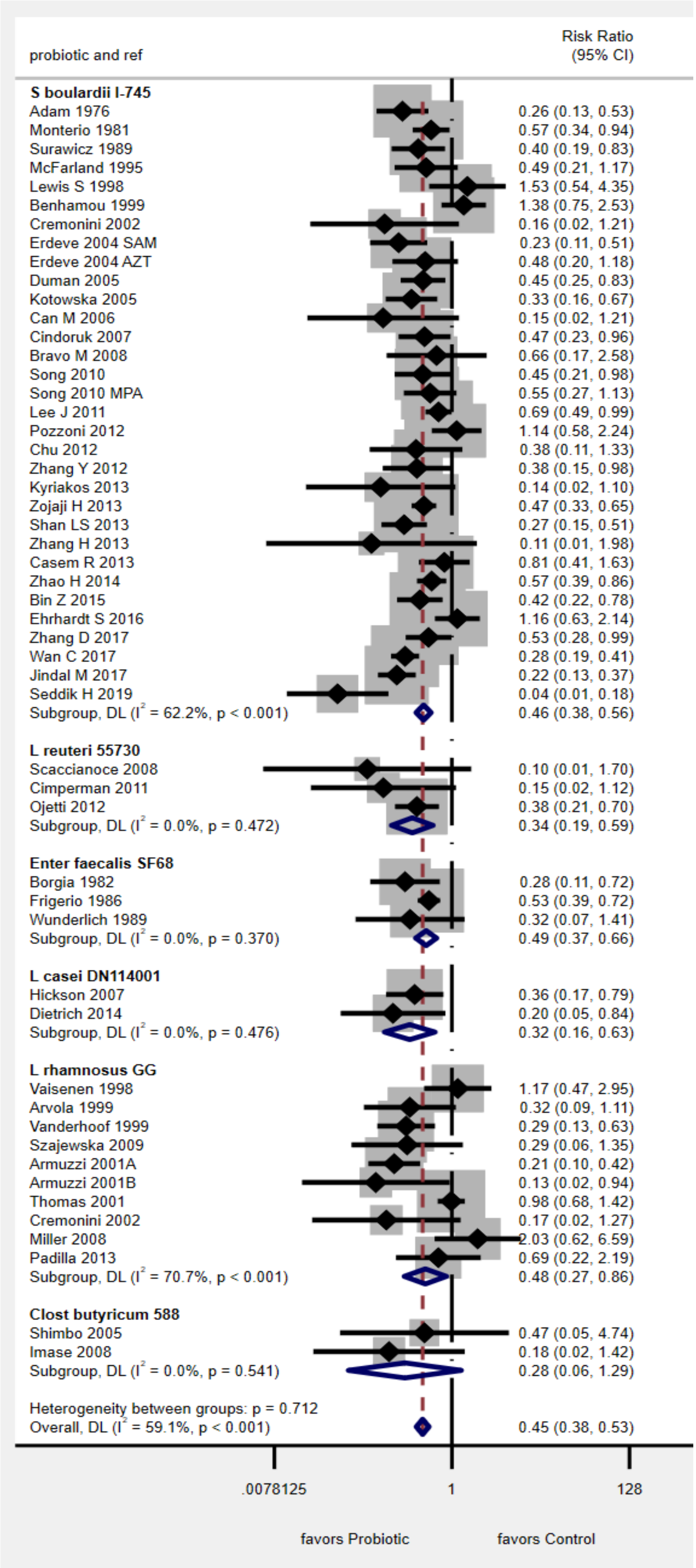

Figure S1. Forest plot of single-strain probiotics for the prevention of antibiotic-associated diarrhea (AAD). Abbreviations: CI, Confidence Interval; *L. casei, Lactobacillus casei*; *L. reuteri, Lactobacillus reuteri*; *L. rhamnosus, Lactobacillus rhamnosus*; *Clost. butryicum, Clostridium butyricum*; *Enter.*

faecalis, Enterococcus faecalis; S. boulardii, Saccharomyces boulardii I-745; RR, Relative Risk. Adapted and updated from: 21, 22, 54-56.

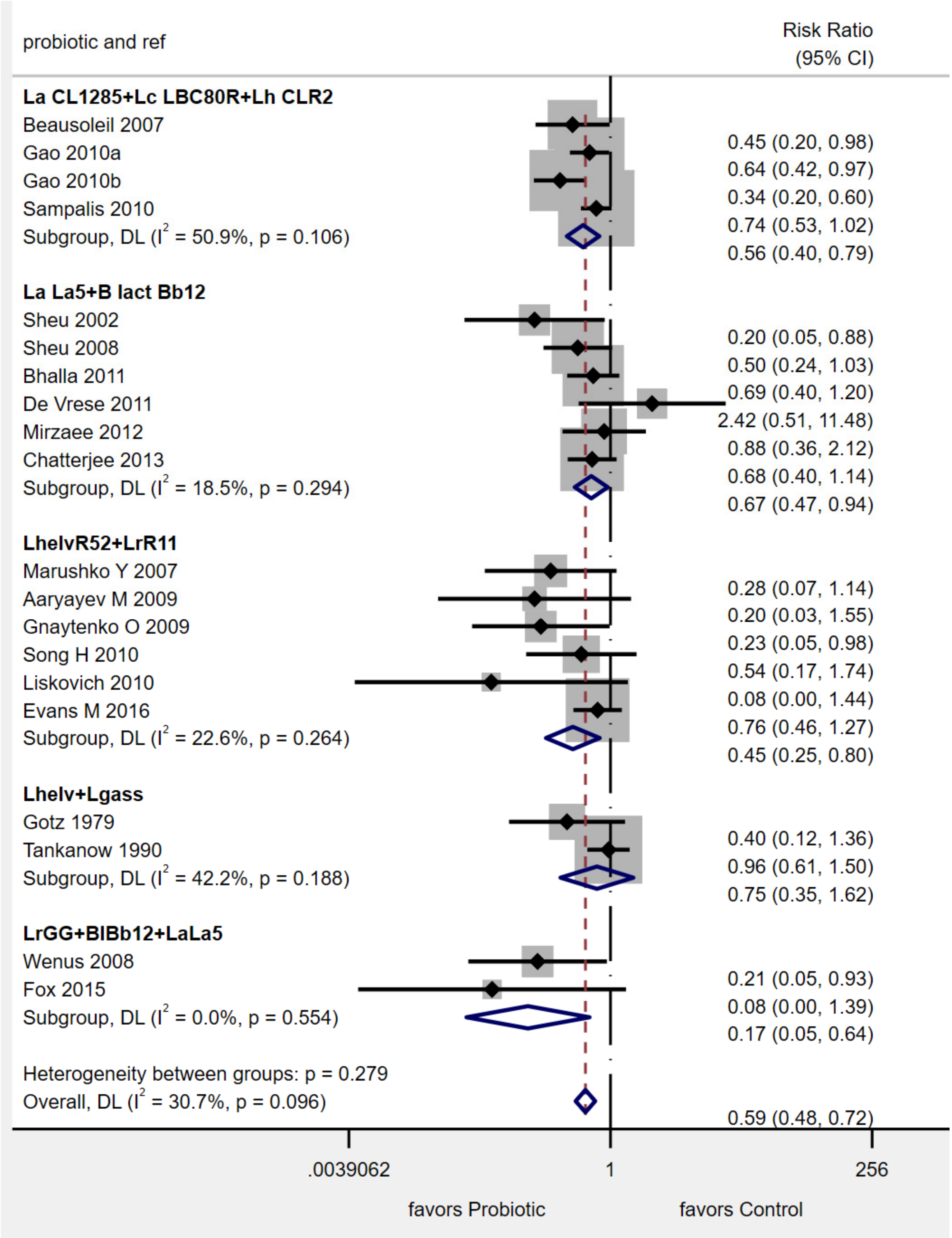

Figure S2. Forest plot of multi-strain probiotic mixtures for the prevention of AAD. **Abbreviations**: C.I., Confidence Interval; La CL1285+Lc LBC80R+Lr CLR2, *Lactobacillus acidophilus CL1285 + Lacticaseibacillus casei CL1285 + Lacticasebacillus rhamnosus CLR2, 'Bio-K+'*; La La5+B lact Bb12, *Lactobacillus acidophilus* La5 + *Bifidobacterium lactis Bb12;* Lhelv R52+Lr R11, *Lactobacillus helveticus* R52 + *Lacticasebacillus rhamnosus R11;* L helv+L gass, *Lactobacillus helveticus + Lactobacillus gasseri*, 'Lactinex'; LrGG+BlBb12+ LaLa5, *Lacticasebacillus rhamnosus* GG+ *Bifidobacterium lactis* Bb12+ *Lactobacillus acidophilus* La5; RR, Relative Risk. Adapted and updated from: 21, 22, 54-56.
